# Intestinal Emphysema and Gut Bacterial Microbiota Composition

**DOI:** 10.3390/microorganisms12050981

**Published:** 2024-05-13

**Authors:** Jasmine Hattab, Alfonso Rosamilia, Chiara Guarnieri, Domenico Sciota, Giuseppe Marruchella, Pietro Giorgio Tiscar

**Affiliations:** 1 Department of Veterinary Medicine, University of Teramo, SP18 Piano d’Accio, 64100 Teramo, Italy; jhattab@unite.it (J.H.); dsciota@unite.it (D.S.); gmarruchella@unite.it (G.M.); 2 Istituto Zooprofilattico Sperimentale della Lombardia e dell’Emilia-Romagna “Bruno Ubertini” (IZSLER), 25124 Brescia, Italy; alfonso.rosamilia@izsler.it; 3 Local Health Authority (ASL), 41121 Modena, Italy; c.guarnieri@ausl.mo.it

**Keywords:** pneumatosis cystoides intestinalis, intestinal emphysema, 16S rRNA gene sequencing, pig

## Abstract

*Pneumatosis cystoides intestinalis*, or intestinal emphysema, is a condition characterized by the presence of multiple cystic structures within the gut wall and on the serosal surface of the intestine. Intestinal emphysema represents an accidental finding in swine, although it can be clinically relevant in humans. Its etiology is unknown, and many theories have been proposed. Among them, a bacterial etiology is considered the most likely. Therefore, in this study, the V3-V4 region of the 16S rRNA gene was sequenced from 19 swine ileal tracts, 12 with intestinal emphysema and 7 without lesions, to detect a possible bacterial agent. In parallel, prevalence was estimated. *Escherichia*–*Shigella* (13.15%), *Clostridium_sensu_stricto_1; s__uncultured_bacterium* (7.09%), and *Fusobacterium; s_uncultured bacterium* (6.60%) were the most abundant species identified. No statistically relevant differences were observed between the pathological and physiological groups. Prevalence ranged from 1.25 to 5.12% depending on the batch. Our results suggest that the gut wall bacterial microbiota greatly match the normal gut microbiota, and that the etiological agent of intestinal emphysema may be (1) undetectable due to the chronicity of the lesions, (2) not considered statistically relevant in comparing the two groups (*p* < 0.05) and likewise in causing lesions, and (3) undetectable due to contamination. Regarding prevalence, the condition is moderately frequent.

## 1. Introduction

*Pneumatosis cystoides intestinalis*, or intestinal emphysema, is a disease characterized by the presence of multiple cystic structures within the gut wall and on the serosal surface of the intestine. The cysts are delimited by thin walls and filled with gas, and their size ranges from millimeters to centimeters. The small intestine is the most frequent location of intestinal emphysema, although it can also be observed in the large intestine and on the mesentery and regional lymph nodes. Microscopically, the cysts are thought to be ectatic lymphatic vessels located in the lamina propria, submucosa, muscularis mucosa, subserosa, and mesentery and mesenteric lymph nodes. A mixed inflammatory infiltrate is often associated with cystic lesions [1].

To date, intestinal emphysema has been recorded in cats [2], rabbits [3], rhesus macaques (*Macaca mulatta*) [4], dogs [5], manatees [6], and ferrets (*Mustela putorius furo*) [7]. Nevertheless, this condition is reported primarily in pigs, in which it is considered an incidental finding mainly observed in weaned pigs and at slaughter [1]. In animals, intestinal emphysema is not associated with clinical disease [8]. Pneumatosis cystoides intestinalis has also been reported in humans, with outcomes ranging from benign to life-threatening, leading to bowel ischemia and necrosis in some cases [9]. In humans, pneumatosis cystoides intestinalis can be primary (or idiopathic) or secondary to pathological conditions such as intestinal necrosis, intestinal obstruction, increased mucosal permeability, mucosal disruption, and pulmonary disease [10].

The root causes have not yet been confirmed, and, to date, two main theories have been proposed. The first suggests that increased intraluminal pressure such as an obstruction may cause gas to leak from the gut lumen into the serosal wall and form cysts. The other hypothesis proposes that bacteria is the origin of the gas filling the cystic formations. According to this theory, the bacteria would reach serosa due to increased mucosal permeability [11]. The incidence of intestinal emphysema is unknown in humans, although the increasing use of CT may help fill this gap [12]. Limited data are available on pigs, and little focus has been placed on the topic. Nevertheless, pigs can represent a model to understand the pathogenesis of intestinal emphysema in other species as well, not least in humans. In this regard, slaughter provides a useful vantage point to outline the real prevalence and the possible causes of the disease.

Recently, metagenomic sequencing has offered great support in revealing the pathogenetic mechanisms of intestinal inflammatory disease. Thus, the aim of the present work was to characterize the microbial communities of the intestinal wall of pigs with and without intestinal emphysema lesions.

## 2. Materials and Methods

### 2.1. Sampling

Samples were collected in a high-production slaughterhouse located in northern Italy.

An ileum tract was collected from each sampled pig. In total, 12 pigs showing pneumatosis cystoides intestinalis lesions and 7 pigs showing no lesions were sampled. The sampling was performed by a skilled veterinarian wearing clean gloves and using a 95 °C sterilized knife. The knife was sterilized between different samplings. The samples were placed in 50 mL Falcons (Securlab, Rome, Italy), and the samples were frozen at −20 °C. Prevalence was assessed concurrently with the sampling, and prevalence within 12 swine batches was assessed.

### 2.2. DNA Extraction

A fragment of the external wall of the intestine (20 μg) was taken with a sterile scalpel from each frozen sample. Total genomic DNA was extracted under a laminar flow cabinet. A commercial kit for DNA isolation was used according to the manufacturer’s instructions (Exgene™ Stool DNA mini, Seoul, Republic of Korea) and stored at −20 °C until use. DNA concentration was assessed with a Qubit fluorometer (Invitrogen, Carlsbad, CA, USA), and samples were normalized at 10 ng/µL concentration.

### 2.3. 16S rRNA Sequencing

The V3–V4 region of the 16S rRNA gene was amplified using the following primers: F, 5′-CCTACGGGNGGCWGCAG-3′ and R, 5′-GACTACHVGGGTATCTAATCC-3′. Primers were modified with forward and reverse overhangs (forward overhang: 5′-TCGTCGGCAGCGTCAGATGTGTATAAGAGACAG-[locus specific sequence]; reverse overhang: 5′-GTCTCGTGGGCTCGGAGATGTGTATAAGAGACAG-[locus specific sequence]) necessary for dual index library preparation. For more details, see the Illumina MiSeq protocol (16S Metagenomic Sequencing Library protocol n. 15,044,223 Rev. B). Sequencing was performed on an Illumina MiSeq (Illumina, San Diego, CA, USA) using a 2 × 300 flow cell V3 chemistry.

### 2.4. Data Analysis

Bacterial microbiota analysis was performed in QIIME 2 2023.5 [13]. A q2-demux plugin was used to demultiplex raw sequences. A quality filter was applied by means of the q2-demux plugin, and denoising was carried out with DADA2 via q2-dada2 [14]. The amplicon sequence variants (ASVs) were then aligned via q2-alignment with MAFFT [15]. The aligned sequences were used to produce an approximately maximum-likelihood phylogenetic tree with FastTree2 via q2-phylogeny [16]. Alpha diversity metrics, namely Chao1, Faith’s Phylogenetic Diversity, Evenness, Observed Features, and the Simpson and Shannon Indexes, were used [17,18,19,20,21]. Beta diversity metrics were estimated to assess differences between groups A, B, and C. In particular, weighted UniFrac, unweighted UniFrac, Jaccard distance, and Bray–Curtis dissimilarity were obtained using q2-diversity [22,23,24,25]. All the of the alpha and beta diversity indexes were computed based on the genus level. Silva v138.1 was used as a reference for taxonomic annotation of ASVs [26,27]. Classification of the reads had 0.96 precision to the genus level, a recall of 0.93, and an F-measure of 0.95. Statistical computing and visualization were performed in the R v4.1 environment [28]. A permutational multivariate analysis of variance (PERMANOVA) test was used to evaluate differences in gut bacterial communities between groups based on 1000 permutations [29]. Results were considered statistically relevant when the *p*-value was below 0.05.

## 3. Results

### 3.1. Sequencing Results and External Gut Wall Communities

**Figure 1:**
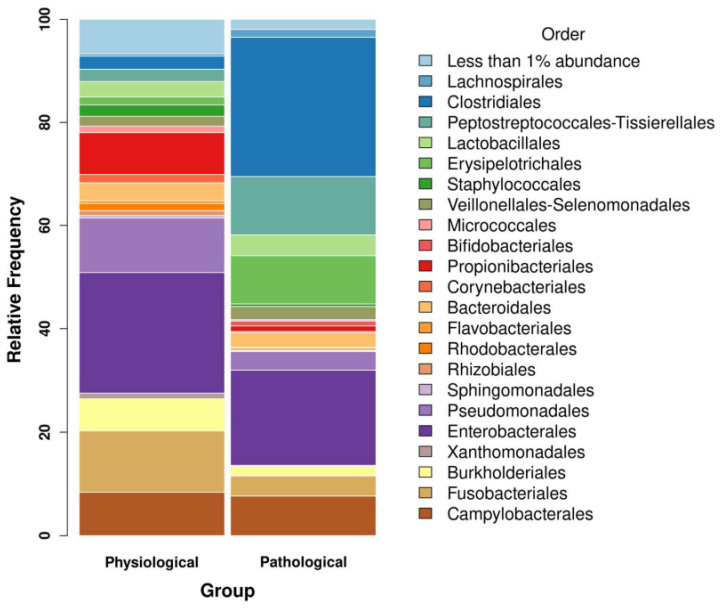
Bar plot representing gut wall bacterial communities of pigs with and without intestinal emphysema lesions (pathological and physiological groups, respectively) at the order level. The orders of physiological and pathological groups with highest relative abundance are shown. Only orders with relative abundance >1 are shown singularly.

In total, 19 samples were analyzed. The samples were divided depending on the presence or absence of lesions compatible with pneumatosis cystoides intestinalis. The groups consisted of 12 and 9 samples, respectively. Overall, 2309 sequences were identified, with a total frequency of 784,991. The maximum and minimum length was equal to 429 and 273 base pairs, respectively. The mean length was 415,52 base pairs (SD 18.03). Enterobacterales were the most abundant order (20,21%), followed by Clostridiales (18.01) and Peptostreptococcales–Tssierellales (8.02%) (Figure 1).

**Figure 2:**
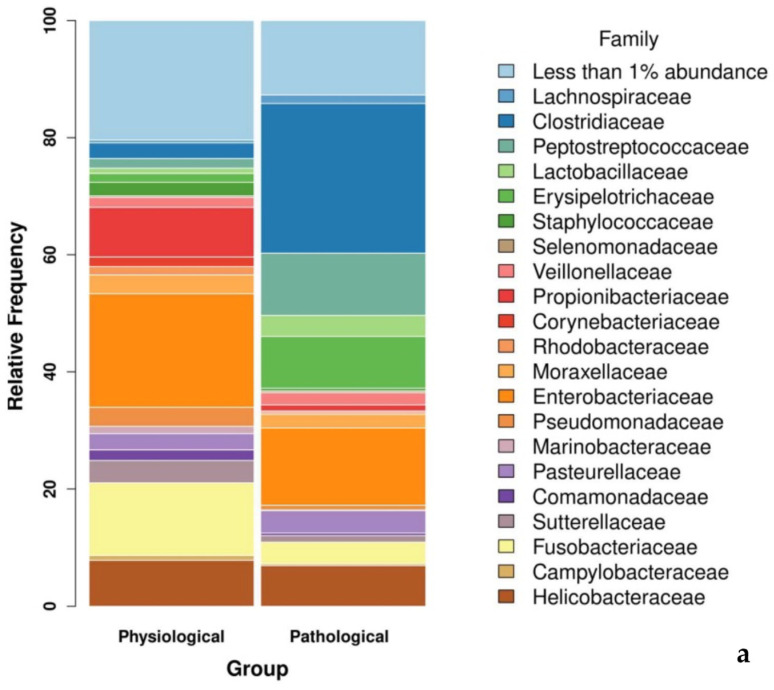

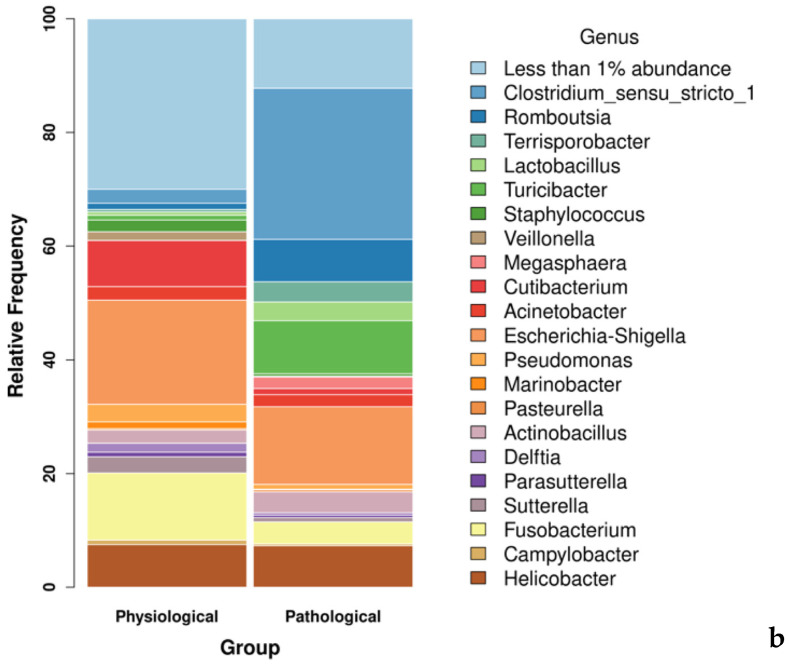
Bar plot representing gut wall bacterial communities of pigs with and without intestinal emphysema lesions (pathological and physiological groups, respectively) at the family and genus level: (**a**) families of the physiological and pathological groups with the highest relative abundance are shown; (**b**) genera of the physiological and pathological groups with the highest relative abundance are shown. Only orders with relative abundance >1 are shown singularly.

Genera and species, whenever identified, with higher relative abundance were *Escherichia*–*Shigella* (13,15%), *Clostridium_sensu_stricto_1; s__uncultured_bacterium* (7.09%), and *Fusobacterium; s_uncultured bacterium* (6.60%) (Figure 2).

### 3.2. Alpha Diversity

**Figure 3:**
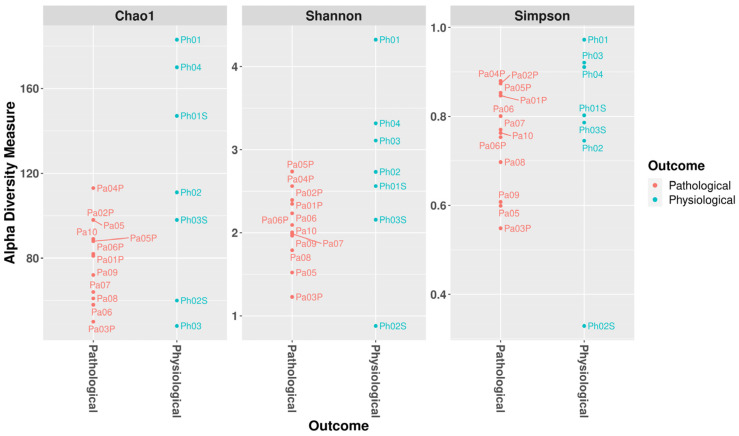
Alpha diversity of the gut wall bacterial communities of pigs. Alpha diversity of the gut bacterial communities of pigs with and without intestinal emphysema lesions (pathological and physiological groups, respectively) according to Chao1, Shannon, and Simpson’s indexes calculated at the genus level.

The Chao1, Shannon, and Simpson indexes were used to assess alpha diversity within each sample (Figure 3).

The evenness, Faith phylogenetic diversity, observed features, and Shannon indexes were used to determine phylogenetic dissimilarity between the two groups, yielding no statistically relevant result (Table 1).

Phylogenetic dissimilarities showed no statistically significant difference between the pathological and physiological groups of samples.

### 3.3. Beta Diversity

**Figure 4:**
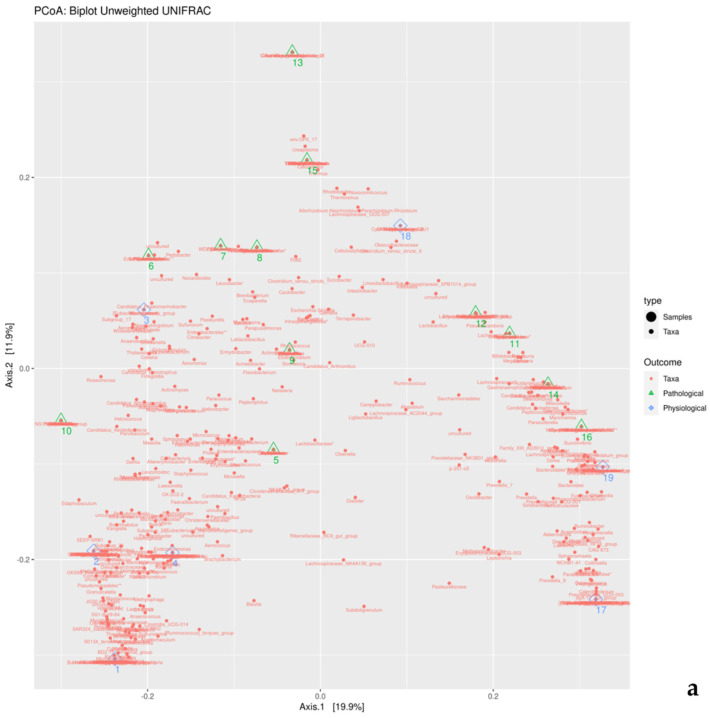

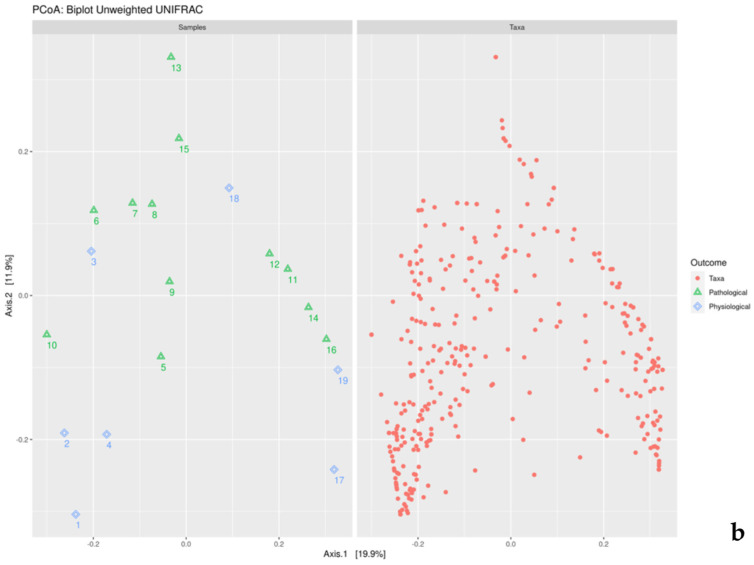
Principal coordinates analysis (PCoA) biplot of the unweighted UniFrac distance matrix generated from taxon abundances showing patterns of beta diversity for gut wall communities of pigs with and without intestinal emphysema lesions (pathological and physiological groups, respectively): (**a**) superimposed taxa distribution and samples; (**b**) separated taxa distribution and samples.

Bray–Curtis dissimilarity and Unweighted UniFrac were used to assess beta diversity with PERMANOVA. Principal coordinates analysis of Unweighted UniFrac was performed (Figure 4).

Pairwise PERMANOVA was performed to compare the unweighted UniFrac results of the two groups, yielding a *p*-value equal to 0.235764.

ANCOM was also performed in order to find differences between the pathological and physiological groups, and no significant features were found.

### 3.4. Prevalence

Prevalence was computed along the slaughter chain in 12 batches of pigs. Excel was used for the calculation (Table 2).

## 4. Discussion

The sequencing of a large number of data, combined with bioinformatics, can lead to the identification of outbreaks, to the tracing of disease transmission, and to the identification of epidemiological dynamics and etiological agents [30]. In the present case, the aim was to identify an etiological agent or a set of microbes that may predispose pigs to the condition under study. In the present work, a metagenomic approach was attempted, with the aim of sequencing all the bacterial genomes found in the samples, both with and without lesions, and comparing the results. No statistically relevant differences were observed between the relative abundance values within the two groups, at all the taxonomic levels of the identified bacterial genomes. The taxonomic composition of the bacterial genomes found in the samples greatly match the composition of the swine intestinal bacterial microbiota found in previous studies. In particular, there was a relevant presence of coliforms and clostridia. *Escherichia*–*Shigella* is reported as the most abundant genus in the ileum of adult healthy pigs, and it is therefore considered normal. Likewise, *Clostridium_sensu_stricto1* is among the most abundant genera found in the same tracts [31]. Fusobacteria are also among the most abundant genera in the ileum of healthy pigs according to McCormack and colleagues [32]. Nevertheless, relative abundance may not be the best indicator to identify a potential pathogen. In fact, its abundance may be lower than the threshold considered statistically significant due to the macrophages’ mechanism of action [33]. Nonetheless, a small amount of genomic material should have been sequenced. No traces of pathogens known for causing granulomatous or necrotizing enterocolitis were detected. Specifically, mycobacteria, *Nocardia*, *Lawsonia intracellularis*, *Brachyspira hyodisenteriae*, and *Salmonella enterica* were not observed [34,35,36]. *Mycobacterium* sp. was found in one physiological sample (0.133%), and it was therefore not considered a possible agent of pneumatosis. *Clostridium perfringens* was found among the species of clostridia observed in the samples, but it was recorded only in six samples, both healthy and pathological, with a relative abundance ranging from 0.018% to 0.673%. Pneumatosis cystoides intestinalis lesions are chronic; therefore, the original cause of the lesions may be difficult to find, since the microorganism responsible for the damage may already have been destroyed or reduced in number by the immune system [33]. On the other hand, the main risk for the metagenomic approach is contamination. In fact, it poses severe difficulties to sampling in a sterile way, particularly in a contaminated environment such as a slaughter chain. It represents a limitation of this study, in which the sampling was carried out in the cleanest way possible, avoiding unnecessary contact with surfaces and using clean gloves and sterile knives. Nonetheless, any contamination of the sample surfaces cannot be excluded and could have influenced the final results. Lastly, the presence of another kind of etiological agent, such as a viral or a fungal agent, cannot be excluded, and further investigation may help clarify the role of such etiologies.

Regarding prevalence, the literature does not provide data on the prevalence in swine. In humans, prevalence ranges from 0.8 to 38,5%, with 43,5% suffering from comorbidity [37]. The data collected in this study revealed that pneumatosis cystoides intestinalis in swine ranges from 1.25 to 5.12% depending on the batch. Therefore, the condition is moderately frequent, yet it has received little attention. This could be because this condition seems to be benign in pigs. However, in humans, pneumatosis cystoides intestinalis has been linked to severe symptoms in some forms [38].

To date, few attempts have been made to clarify pneumatosis cystoides intestinalis’ etiology [39,40]. Specifically, lesions referrable to pneumatosis were observed during necropsy in a gnotobiotic pig experimentally infected with an enteropathogenic strain of *Escherichia coli.* Therefore, *E. coli* infection was considered to play a role in the development of the lesions [39]. Another case report of pneumatosis in a pig suggests that abdominal blunt trauma is the cause of the lesions. According to the authors, such trauma may have caused increased permeability in the intestinal mucosa and therefore the translocation of gas into the gut wall, as also reported in humans [41,42]. In another study, arteries, veins, and lymphatics were experimentally occluded in neonatal piglets. This procedure reproduced intestinal emphysema lesions, endorsing the theory that increased permeability of the mucosa may generate the characteristic lesions [43]. According to a recent review, the secondary causes in humans can be linked to various conditions involving the gut, but in many cases, the etiology is unknown [12].

## 5. Conclusions

This study provided an outline of the bacterial microbiota of the external gut wall. The bacterial composition of the communities inhabiting this district is mainly *Escherichia*–*Shigella*, *Clostridium_sensu_stricto_1; s__uncultured_bacterium*, and *Fusobacterium; s_uncultured bacterium* (6.60%). This structure strongly resembles that of normal gut bacterial microbiota. No potential etiology for pneumatosis cystoides intestinalis was identified, nor were pathogens found. Further studies, such as research involving non-bacterial etiologies, should be performed in order to determine the possible cause of this condition.

## Figures and Tables

**Table 1:** Phylogenetic dissimilarity *p*-values for each index comparison between the two groups.

Index	*p*-Value
Evenness	0.12819
Faith phylogenetic dissimilarity	0.090969
Observed features	0.271688
Shannon	0.062979

**Table 2:** Prevalence of pneumatosis cystoides intestinalis in 12 batches of pigs at slaughter.

Batch	Number of Slaughtered Pigs	Number of Pigs with Intestinal Emphysema	Prevalence
*1*	130	2	2.6
*2*	128	2	2.56
*3*	128	3	3.84
*4*	128	4	5.12
*5*	126	1	1.26
*6*	132	2	2.64
*7*	130	2	2.6
*8*	130	1	1.3
*9*	130	3	3.9
*10*	125	1	1.25
*11*	125	3	3.75
*12*	130	3	3.9

## Data Availability

Data are contained within the article.
